# Study on establishment and mechanics application of finite element model of bovine eye

**DOI:** 10.1186/s12886-015-0073-4

**Published:** 2015-08-13

**Authors:** Yan-Hui Cui, Ju-Fang Huang, Si-Ying Cheng, Wei Wei, Lei Shang, Na Li, Kun Xiong

**Affiliations:** Department of Anatomy and Neurobiology, Morphological Sciences Building, Central South University, 172 Tongzi Po Road, Changsha, Hunan 410013 China; Eight-year clinical medicine, 2011 grade, Xiangya Medical School, Central South University, Changsha, Hunan 410013 China; Laboratoire de Biomécanique Appliquée, MRT24 IFSTTAR-Aix-Marseille Université, Bd. P. Dramard, Faculté de Medecine secteur-Nord, Marseille, 13916 France; Radiology Department, Third Xiangya Hospital, Central South University, 138 Tongzi Po Road, Changsha, Hunan 410013 China

**Keywords:** High increased intraocular pressure, Sclera, Lamina cribrosa, Finite element modeling

## Abstract

**Background:**

Glaucoma mainly induced by increased intraocular pressure (IOP), it was believed that the pressure that wall of eyeball withstands were determined by material properties of the tissue and stereoscopic geometry of the eyeball. In order to study the pressure changes in different parts of interior eyeball wall, it is necessary to develop a novel eye ball FEM with more accurate geometry and material properties. Use this model to study the stress changes in different parts of eyeball, especially the lamina cribrosa (LC) under normal physiological and pathological IOP, and provide a mathematical model for biomechanical studies of selected retinal ganglion cells (RGCs) death.

**Methods:**

(1) Sclera was cut into 3.8-mm wide, 14.5-mm long strips, and cornea was cut into 9.5-mm-wide and 10-mm-long strips; (2) 858 Mini BionixII biomechanical loading instrument was used to stretch sclera and cornea. The stretching rate for sclera was 0.3 mm/s, 3 mm/s, 30 mm/s, 300 mm/s; and for cornea were 0.3 mm/s and 30 mm/s. The deformation-stress curve was recorded; (3) Naso-temporal and longitudinal distance of LC were measured; (4) Micro-CT was used to accurately scan fresh bovine eyes and obtain the geometrical image and data to establish bovine eye model. 3-D reconstruction was performed using these images and data to work out the geometric shape of bovine eye; (5) IOP levels for eyeball FEM was set and the inner wall of eyeball was used taken as load-bearing part. Simulated eyeball FE modeling was run under the IOP level of 10 mmHg, 30 mmHg, 60 mmHg and 100 mmHg, and the force condition of different parts of eyeball was recorded under different IOP levels.

**Results:**

(1) We obtained the material parameters more in line with physiological conditions and established a more realistic eyeball model using reversed engineering of parameters optimization method to calculate the complex nonlinear super-elastic and viscoelastic parameters more accurately; (2) We observed the following phenomenon by simulating increased pressure using FEM: as simulative IOP increased, the stress concentration scope on the posterior half of sclera became narrower; in the meantime, the stress-concentration scope on the anterior half of scleral gradually expanded, and the stress on the central part of LC is highest.

**Conclusion:**

As simulative IOP increased, stress-concentration scope on the posterior half of sclera gradually narrowed; in the meantime, the stress-concentration scope on the anterior half of sclera gradually expanded, and the stress on the LC is mainly concentrated in the central part, suggesting that IOP is mainly concentrated in the anterior part of the eyeball as it increases. This might provide a biomechanical evidence to explain why RGCs in peripheral part die earlier than RGCs in central part under HIOP.

## Background

Glaucoma is a common blinding eye disease, which is induced by increased intraocular pressure (IOP) leading to problems like optic papilla sag, optic atrophy, visual field defect and loss of retinal ganglion cells (RGCs) [1–3]. The main risk factors of glaucoma include anatomical abnormalities stemming from inter-individual differences, intraocular pressure, local blood supply, genetic factors and refractive errors, *etc.* [4–8]. One study showed that the death of RGCs in glaucoma patients never occurred at the same time. Instead, it is a slow process, in which the subjective visual defects caused by the loss of visual sensitivity in the peripheral part of retina happens earlier than the central part [9]. Animal studies also suggested that the loss of RGCs in the central part occurs later than that of the peripheral part [10, 11]. These phenomena indicated that the damage of different RGCs varies in glaucoma, and the underlying neurobiological and biochemical mechanisms for these differences have been investigated. For example, some reports showed various distribution of excitatory neurotransmitter and neurotrophic factors in different kinds of RGCs [12–20], while others showed that the blood supply was different in different parts of retina [21]. However, it remains unclear whether RGCs in different parts of retina bear the same pressure after increased IOP in glaucoma.

It is believed that the pressure on the wall of eyeball is determined by material properties of the tissue and stereoscopic geometry of the eyeball. Sclera, as one of the most important structures of the wall of eyeball, has been the focus of relative studies, which indicated that it was more likely to develop myopia when scleral creep rate was higher [22–24]. Philips *et al*. studied the sclera of chicken with induced monocular myopia, and found that the axial length change in myopia was mainly caused by the change of scleral creep properties [25], suggesting that the geometry and material properties of eyeball may have significant impact on the physiological and pathological changes of the eye. These studies also pointed out that tissues bearing the load of optic papilla, such as sclera around optic papilla, lamina cribrosa (LC), wall of scleral canal, also bear the pressure caused by IOP. In the past two decades, the FE modeling has been widely utilized in IOP studies. For example, Bellezza *et al*. [26] studied the IOP-induced biomechanical changes of sclera , and Sigal *et al*. [27] proved that FE model had a good consistency with real eyes in geometric shape, elastic mechanical response and viscous mechanical response. Richard *et al*. [28] established a “Universal” FEM of human optic papilla which contained LC using the method by Sigal *et al*. [29]. Sensitivity analysis of the experiment revealed that the thickness of sclera around optic papilla had a strong effect on biomechanics of eyeball, which could influence the responses of LC and altered the displacement of optic papilla under all pressure levels. Due to the anatomical and functional complexity of the eyeball, it is usually difficult to experimentally study the biomechanical responses of eyeball components with detailed optic papilla, sclera and LC under both physiological and HIOP loading. It is therefore necessary to incorporate Finite Element analysis to more accurately study the subtle changes and biomechanical responses of the eyeball.

Previous studies on biomechanical model showed that the mechanical stress on optic papilla induced by increased IOP could trigger a series of problems, eventually leading to the dysfunction and death of RGCs [30]. Although the mechanisms have not been completely investigated, researches have found that LC was a vulnerable area to injury [31–33], suggesting LC-related damage might be the direct cause of RGCs injury. In addition, previous studies [34, 35] showed that scleral materials, geometry and thickness significantly affected the biomechanics of optic papilla. Finite element studies of primates also showed that the sclera played a significant biomechanical role in the biomechanics of optic papilla [36, 37]. However, these studies all used “spherical sclera shell” models with uniform thickness, which over simplified the physiological and anatomical characteristics of sclera. For further studying the effects of increased IOP on function and death of RGCs, it is necessary to analyze stress changes of interior eye wall, such as different stress distribution under normal and increased IOP, or to what extent the heterogeneous thickness of eye tissues will affect stress distribution. To solve the above problems, it is necessary to establish a new FEM based on heterogeneous thickness of eyeball tissues. Therefore, in order to study the pressure changes in different parts of interior eyeball wall, including LC regions, under different IOP, in the present study we established FEMs of healthy bovine eyes, in which the realistic parameters of eyeball, especially the thickness and geometry of sclera were considered.

## Methods

### Sample collection

Forty bovine eyes were collected from Luxi cattle. Twenty of them, including 8 from two-year-old, 8 from three-year-old and 4 from four-year-old bovine, were used for biomechanical experiment. The other 20 eyes, including 8 from two-year-old, 8 from three-year-oldand 4 from four-year-old bovine, were used for LC measurement. The selected cattle with glossy hair, bright and transparent pupil, and spotless cornea, were healthy and met all physiological criteria. Sample collection time: 10–30 minutes after death. Respective mark above orbital cavity and nasal side were made to locate eyeball before sample collection. All experimental procedures used in the present study were approved by Ethics Committee of Xiangya School of Medicine, in accordance with the NIH guidelines for use and care of laboratory animals. All of our eyeballs were purchased from Luxi cattle slaughter house, and we have gotten the permission from the owner.

### Preparation and measurement of sclera and corneal strips

**Fig. 1:**
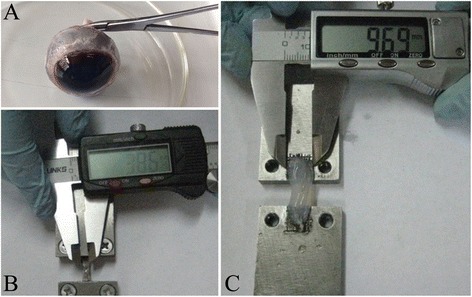
Preparation and measurement of scleral and corneal strips. **a**: The eyeball that was removed and cleaned up; **b**: Measurement of scleral width; **c**: Measurement of corneal width

Tissues and muscles around the eyeball were removed (Fig. 1a), then the eyeball was cut open from sclera-corneal junction, corneal separated from sclera and the intraocular contents were disposed. Retina and choroid were cleaned from the interior sclera. Cautions were taken not to hurt the scleral tissues. Sclera and cornea were then cut into strips. Sclera was cut into strips with an average width of 3.8 mm and length of 14.5 mm which radiated from lamina cribiosa. Cornea was cut into strips with an average width of 9.5 mm and length of 10 mm (Fig. 1b and c). It should be noted that retina and choroid are quite weak and fragile. With a thickness of only about 250 μm and Young's modulus of only 20 kPa [37], retina cannot bear load, thus our experiment only analyzed the material characteristics of load bearing tissues like sclera and cornea.

### Measurement of bovine LC

**Fig. 2:**
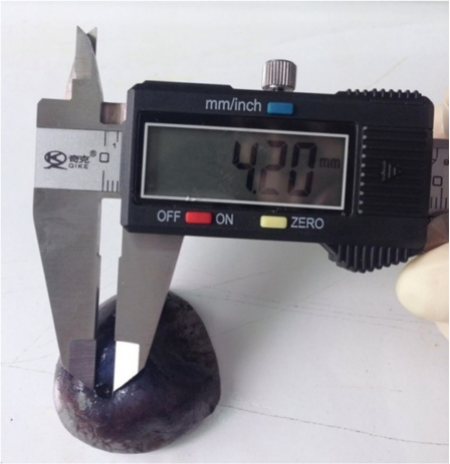
Picture of LC measurement

After cleaning up the tissues and muscles around eyeball and removing the retina and choroid, the eyeball was soaked in distilled water for one hour to remove prelaminar tissues and fix the sclera. Vernier caliper was used to measure the naso-temporal width of LC and the length between top and lower LC (Fig. 2).

### Preparation of biomechanical experiment

**Fig. 3:**
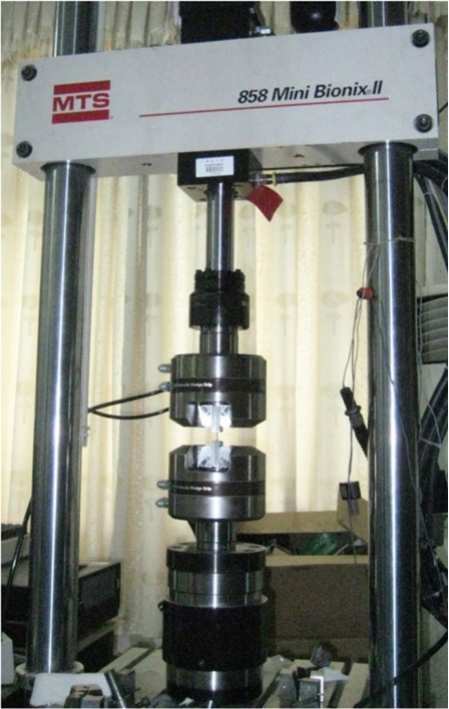
Biomechanical loading picture of eye material

After cutting the cornea and sclera into regular strips, they were fixed between two stainless steel plates (Fig 1b and c). Sandwiched with sample, steel plates were installed onto 858 Mini BionixII (MTS Co., Minneapolis, MN, USA), a biomechanical loading instrument (Fig. 3). The scleral strips were stretched at different speed of 0.3 mm/s, 3 mm/s, 30 mm/s and 300 mm/s, and the corneal strips were stretched at 0.3 mm/s and 30 mm/s. The deformation-force curve was recorded.

### Development of eyeball FE modeling

Micro-CT (ZKKS, Sharp, Osaka, Japan) has been used to accurately scan the fresh bovine eyes, with the slice thickness of 0.07 mm, and segmentation and smoothing of scleral and corneal images from different layers has been finished. Based on these, we did three-dimensional reconstruction using MIMICS (Version 12.0, Materialise Inc., Leuven, Belgium) and finally got the geometry of eyeball with heterogeneous thickness.

We meshed the eyeball geometry using HYPERMESH (Version 10.0, Altair, Troy, MI, USA). The model was divided into three key parts: sclera, cornea and LC. The units had total 14,370 nodes and 19,152 hexahedral elements, and each of elements was 0.7 to 1 mm in size. In order to ensure the accurate operation of the model, we strictly controlled the quality of elements during meshing. The minimum Jacobian Ratio was 0.47 and the minimum angle was 43.53 degrees.

**Fig. 4:**
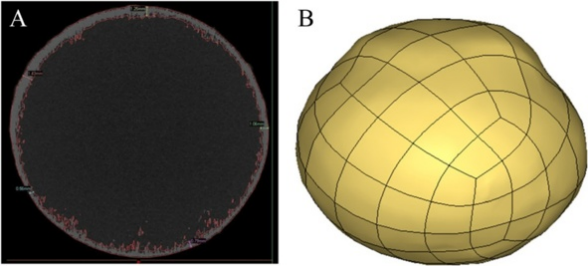
Micro-CT scanning image of bovine eyes and the established FEM model. **a**: Image of Micro-CT scanning of bovine eyes and scleral thickness measurement; **b**: Three dimensional FEM based on bovine eyes

In order to overcome the defects in previous experiments, namely the over simplification of eyes by FEM, such as simplifying the eyeball into a sphere, and scleral thickness into uniform thickness, we segmented 700 transverse sectional images of eyeball (Fig. 4a) and conducted three-dimensional reconstruction (Fig. 4b) based on the pictures from Micro-CT scanning. We accurately measured the thickness of bovine eyeball wall, including sclera and cornea, using MIMICS software and established a three-dimensional FEM of bovine eyes, in which important factors for FE model such as geometry and anatomical structure of eyeballs, scleral and corneal were considered.

### Methods optimization

Most previous studies used linear elastic material when selecting the material parameter of the eyeball model [38–41], however [42], the human body is actually made up of both hyperelastic and nonlinear viscous materials [42]. It performs as hyperelastic material under low speed conditions and its stress–strain curve changes along with the alteration of loading conditions, with high velocity loadings. In order to reflect these two characteristics, our research combines the nonlinear elastic material theory of Ogden [43] with viscoelastic mechanics theory of Christensen [44]. Ogden’s theory takes α and μ as material parameters and can adequately reflect the non-linear stress–strain curve under Quasi static state; Christensen’s theory takes β and G as material parameters and can reflect the mechanical properties that stress and strain change with the alteration of loading velocity, and the mathematic derivation process is as follows:

Based on Ogden’ theory, the energy density equation for incompressible materials can be expressed as follows:

1 $$ W={\displaystyle \sum_{i=1}^3{\displaystyle \sum_{j=1}^n\frac{\mu_j}{\alpha_j}\left({\lambda}_i^{\alpha_j}-1\right)+\frac{1}{2}K{\left(J-1\right)}^2={\displaystyle \sum_{i=1}^3{\displaystyle \sum_{j=1}^n\frac{\mu_j}{\alpha_j}\left({\lambda}_i^{\alpha_j}-1\right)}}}} $$

Where I set as the direction of deformation, J the order for parameter calculation and n can be any integer value from 1 to 8. We only represent three perpendicular directions in this equation.

2 $$ W={\displaystyle \sum_{i=1}^3{\displaystyle \sum_{j=1}^n\frac{\mu_j}{\alpha_j}\left({\lambda}_i^{\alpha_j}-1\right)={\displaystyle \sum_{j=1}^n\frac{\mu_j}{\alpha_j}\left({\lambda}_x^{\alpha_j}+{\lambda}_y^{\alpha_j}+{\lambda}_z^{\alpha_j}-3\right)}}} $$

The following can be derived from equation (1) and (2):

3 $$ W={\displaystyle \sum_{j=1}^n\frac{\mu_j}{\alpha_j}\left({\lambda}_x^{\alpha_j}+2{\lambda}_x^{\frac{-1}{2}{\alpha}_j}-3\right)} $$

Therefore the hyperelastic stress can be expressed as:

4 $$ {\sigma}_{hyper}={\mu}_j\left({\lambda}_x^{\alpha_j}-{\lambda}_x^{\frac{-{\alpha}_j}{2}}\right) $$

Based on the viscoelastic theory of Christensen, the effect of strain rate on the viscoelastic stress can be expressed by a nonlinear viscoelastic all-integral equation:

5 $$ {\sigma}_{ij}={\displaystyle {\int}_0^t{g}_{ijkl}}\left(t-\tau \right)\frac{\partial {\varepsilon}_{kj}}{\partial \tau }d\tau $$

The total stress on the tested material is combined from both hyperelastic part and viscoelastic part, as shown in equation (6):

6 $$ {\sigma}_{total}={\mu}^{*}{\lambda}_x^{\alpha -1}+{\displaystyle \underset{0}{\overset{t}{\int }}{G}^{*}{e}^{-\beta \left(t-\tau \right)}}\frac{\partial \varepsilon }{\partial \tau }d\tau $$

**Fig. 5:**
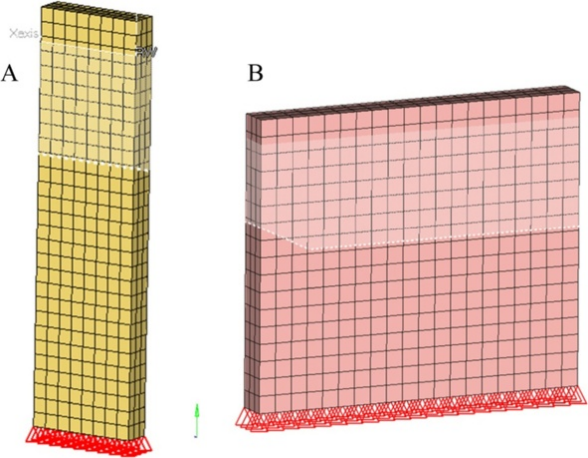
FEM of sclera and cornea. **a**: sclera; **b**: cornea

Perform the reverse engineering of material mechanics on the experimental samples. Firstly, establish the mechanical model of experimental samples (Fig. 5):

Simulate corneal stress based on results of tension experiments and perform the curve-fitting of displacement versus force curve. Obtain hyperelastic-viscoelastic mechanical parameters of materials by using optimal calculation of reverse engineering methods showed as follows. Finish the optimal reverse engineering of scleral and corneal and obtain the materials parameters using Non-dominated Sorting Genetic Algorithm-II (NSGA-II) [45], set α, μ, β, Gas input variables, μ and G stands for hyperelastic modulus and viscoelastic modulus, respectively, measured in units of MPa. β stands for hyperelastic parameter and viscoelastic parameter firstly.

**Fig. 6:**
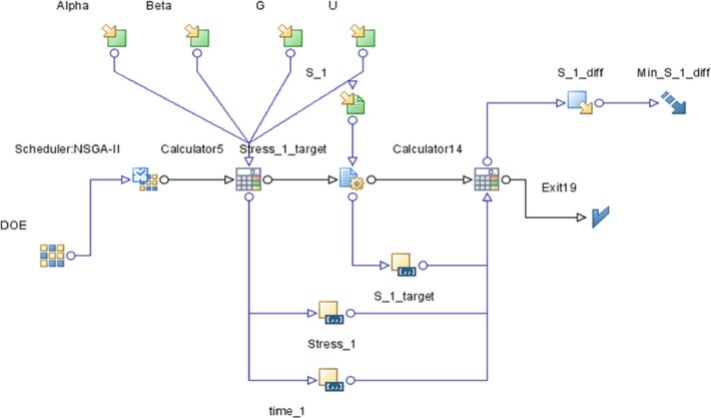
NSGA-II optimiazation Algorithm process design

Secondly, finish the design of experiment (DOE) and define the scope of each input variable (Fig. 6):

Use Mode FRONTIER software (Version 4.0, ESTECO, Trieste, Italy) to optimize four Ogden material models in FEM: parameter α, μ, β and G. Steps are as follows: The upper and lower limits of parameter α, μ, β and G are set initial values according to values recorded in pertinent literature, use Mode FRONTIER to randomly choose parameter values within its ranges as input value, calculate with formula used in theoretical analysis of continuum medium mechanics, take the calculation results with minimum error value as the optimal results. When designing the experiment, use Sobol’s method to randomly select a series of samples as estimated input variables and establish the sample space. Samples were uniformly distributed in the generation region. Maximum Number of Iteration is 100. Repeatedly calls the simulative running of LS-DYNA (LS-DYNA3D 971, LSTC, Livermore, CA, USA) FEM, the number of call can be adjusted according to error range. After finishing the optimization, enter the best estimating parameters as the material parameter into the FEM and perform analog computation using LS-DYNA.

### Mechanical condition settings of IOP

In order to fully simulate the impact of IOP on the inner wall of eyeball, we chose nodes on the inner wall of eyeball as the setting area that bear stress; the direction of IOP was perpendicular to each node. Perform analog computation after setting pressure value at 10 mmHg, 30 mmHg, 60 mmHg and 100 mmHg, where 10 mmHg is the IOP value for normal human eyes; 30 mmHg is the IOP value for low or middle IOP glaucoma [46]; 60 mmHg is the IOP value for HIOP glaucoma [47]; 100 mmHg is the commonly used IOP value for animal glaucoma experiments [48].

## Results

### LC size and corneal and scleral thickness

It is necessary to measure the LC size of bovine eyes since the Micro-CT is not able to show its size and position. Twenty processed fresh bovine eyes have been selected; the LC size and scleral thickness around optic papilla of each sample were measured using a vernier caliper (Table 1). The results are as follows: the naso-temporal length range of LC was: 4.37 to 4.71 mm, the longitudinal length range of LC was: 3.49 to 3.84 mm, range of scleral thickness around optic papilla: 1.55 to 1.86 mm.

**Table 1:** The measurement of size of LC

Age (year)	Naso-temporal width of LC (mm)	Longitudinal length of LC (mm)
2	4.57	3.53
2	4.62	3.7
2	4.46	3.81
2	4.59	3.49
2	4.37	3.66
2	4.62	3.72
2	4.51	3.55
2	4.57	3.6
3	4.62	3.76
3	4.68	3.53
3	4.7	3.78
3	4.62	3.69
3	4.63	3.71
3	4.71	3.74
3	4.63	3.81
3	4.65	3.62
4	4.67	3.51
4	4.56	3.73
4	4.69	3.82
4	4.58	3.84
mean	4.60	3.68
SD	0.081	0.109

### Deformation-force curve

**Fig. 7:**
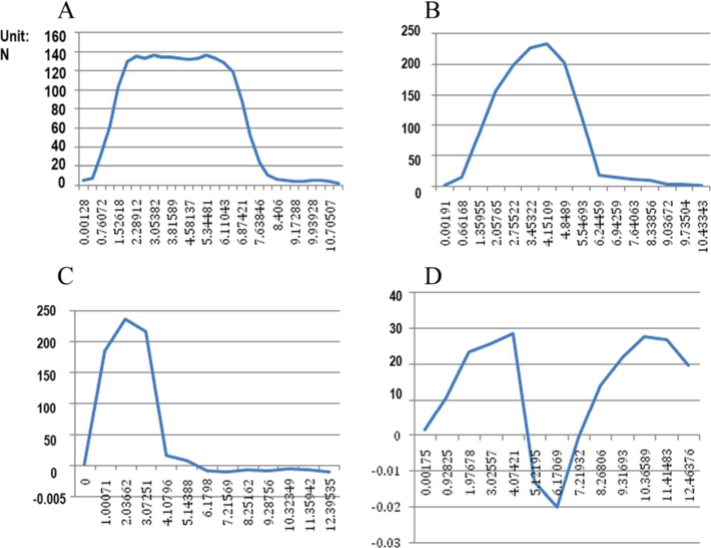
Deformation-stress curve of sclera under tensile rate of 0.3 mm/s-300 mm/s. **a**: 0.3 mm/s; **b**: 3 mm/s; **c**: 30 mm/s; **d**: 300 mm/s

**Fig. 8:**
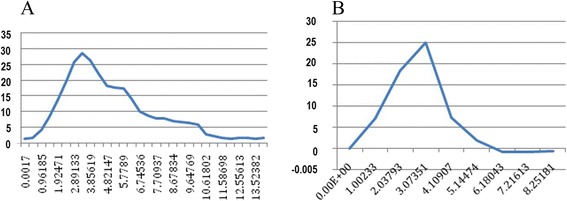
Deformation-stress curve of cornea under tensile rate of 0.3 mm/s-300 mm/s. **a**: 0.3 mm/s; **b**: 30 mm/s

Figures 7 and 8 showed the measured displacement versus force curves under different tensile velocity. The deformation-force curve with lowest speed was at quasi-static state in this loading, and the hyper-elastic material parameters α and μ could be obtained by optimal NSGA-II reverse engineering. In order to obtain a more accurate viscoelastic parameter, we performed tension tests at multiple strain rates and the obtained data were used for calculation of viscoelastic material parameter β and G (Table 2). The results showed that the stress was smallest under low speed (0.03 mm/s) condition; the maximum force was 16 N from the obtained experimental curve; when the stretching speed is 300 mm/s, the maximum force reached 30 N. This phenomenon defined the time-dependent viscoelastic properties of scleral material.

**Table 2:** Hyperelastic parameters and viscoelastic parameters after optimization

Location	α	μ (MPa)	β	G (MPa)
Cornea	8.1	0.675	0.13	1.641
Sclera	27.7	0.774	0.93	1.8

### Hyperelastic parameters and viscoelastic parameters after optimization

The reverse optimization method of FEM that we used was based on optimization method simulated by small FEM. Compared with the traditional curve fitting methods, this method performed specimen-specific optimization therefore can more accurately obtain complicated nonlinear hyperelastic parameters and viscoelastic parameters at the same time. NSGA-II optimization method was widely adopted by researchers in the field of FEM optimization [49] and has made significant contributions in improving the calculation accuracy in biomechanical field [50]. By applying this method to the establishment of bovine eye FEM, we not only obtained more accurate material parameters, as shown in Table 2, but also established a more realistic eyeball model and provided a reliable basis to truly simulate the biomechanical responses when IOP was elevated.

### FEM-simulated IOP change

**Fig. 9:**
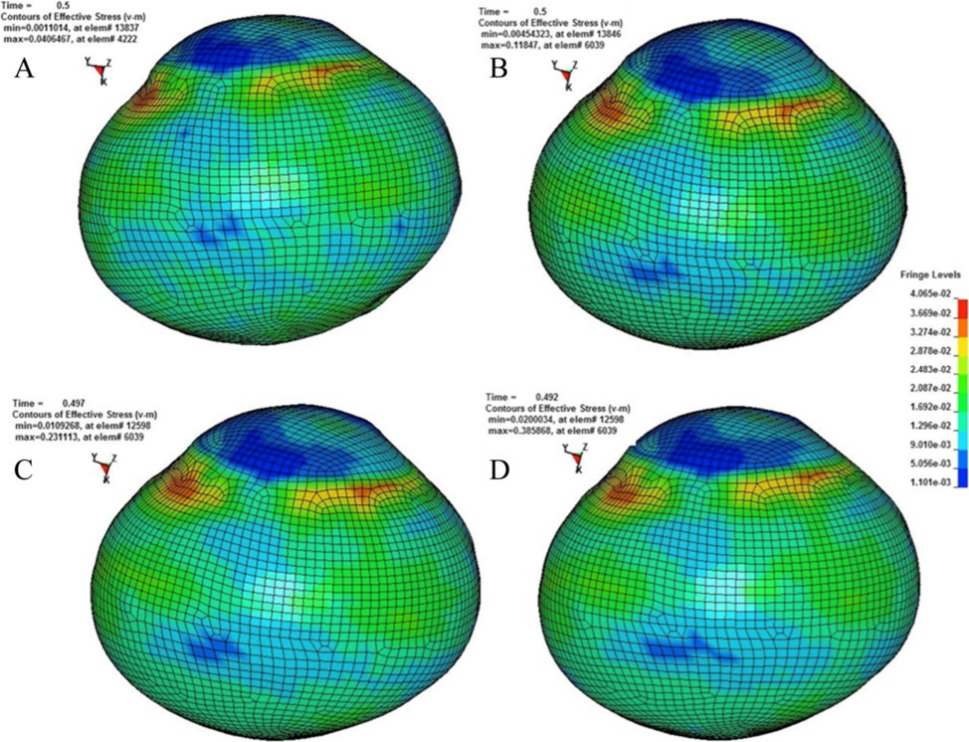
Equivalent strain nephogram of eyeball. **a**: 10 mmHg; **b**: 30 mmHg: **c**: 60 mmHg; **d**: 100 mmHg

**Fig. 10:**
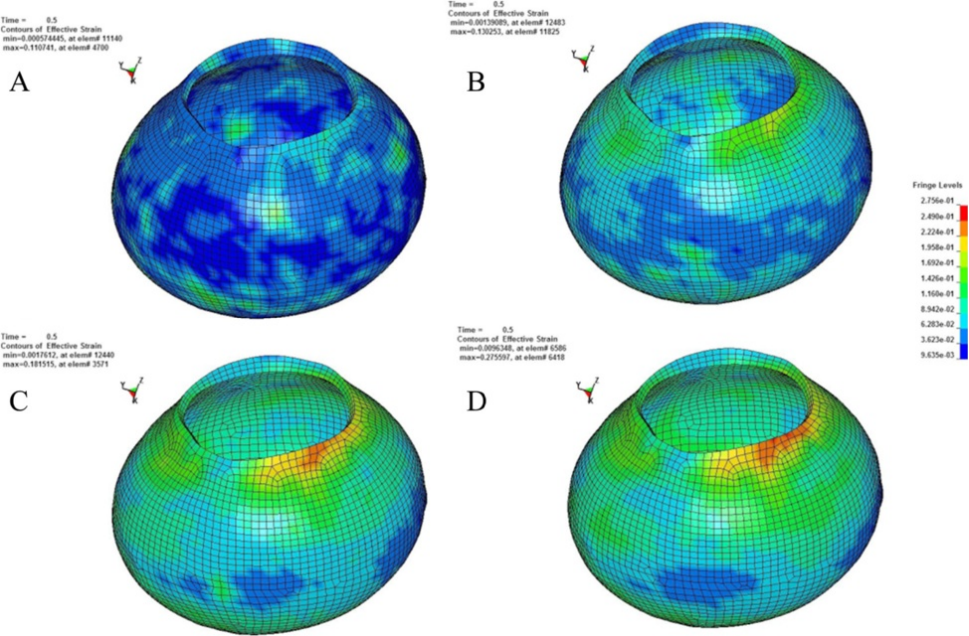
Equivalent strain nephogram of sclera. **a**: 10 mmHg; **b**: 30 mmHg; **c**: 60 mmHg; **d**: 100 mmHg

**Fig. 11:**
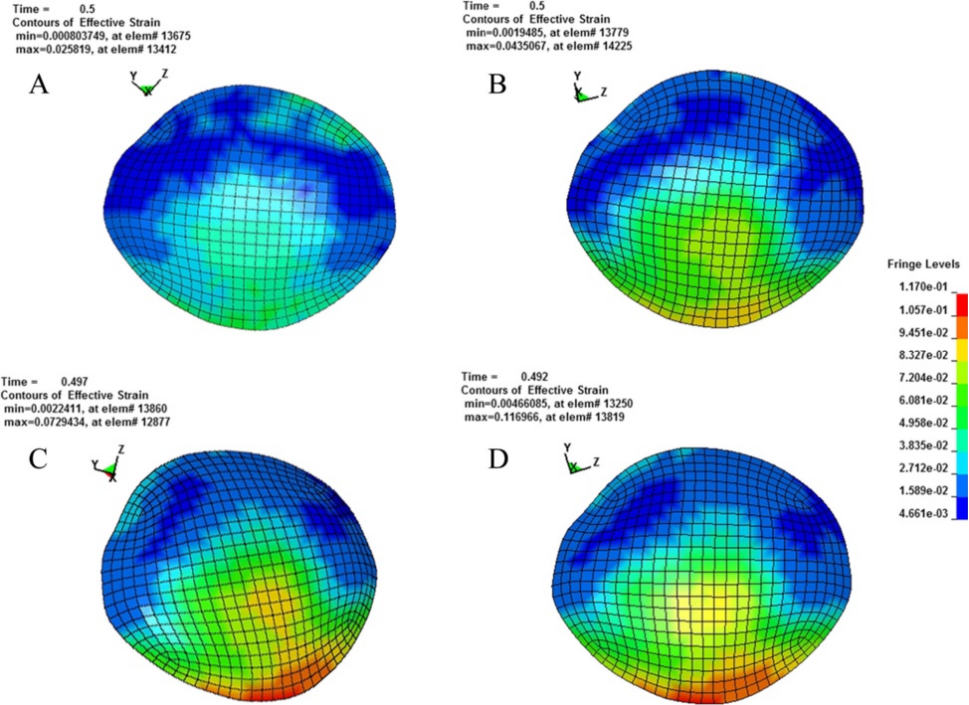
Equivalent strain nephogram of cornea. **a**: 10 mmHg; **b**: 30 mmHg; **c**: 60 mmHg; **d**: 100 mmHg

**Fig. 12:**
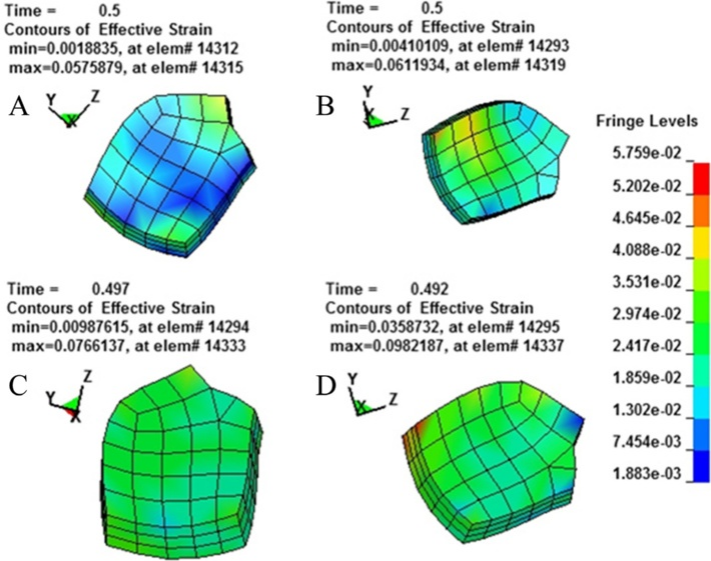
Equivalent strain nephogram of LC. **a**: 10 mmHg; **b**: 30 mmHg; **c**: 60 mmHg; **d**: 100 mmHg

**Fig. 13:**
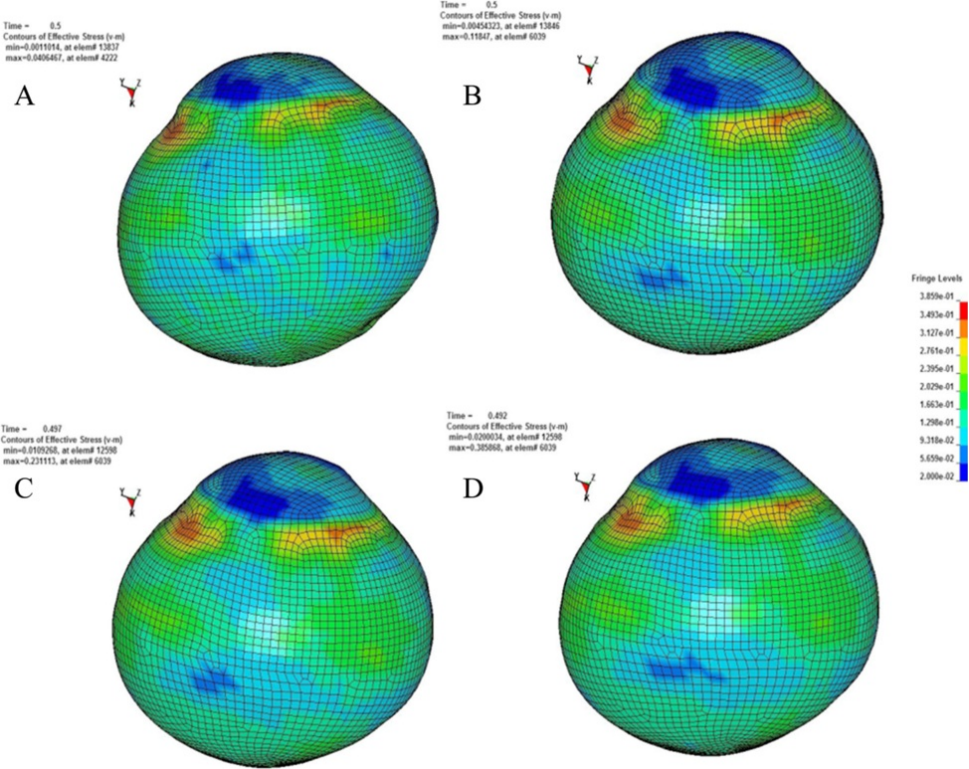
Equivalent stress nephogram of eyeball. **a**: 10 mmHg; **b**: 30 mmHg; **c**: 60 mmHg; **d**: 100 mmHg

**Fig. 14:**
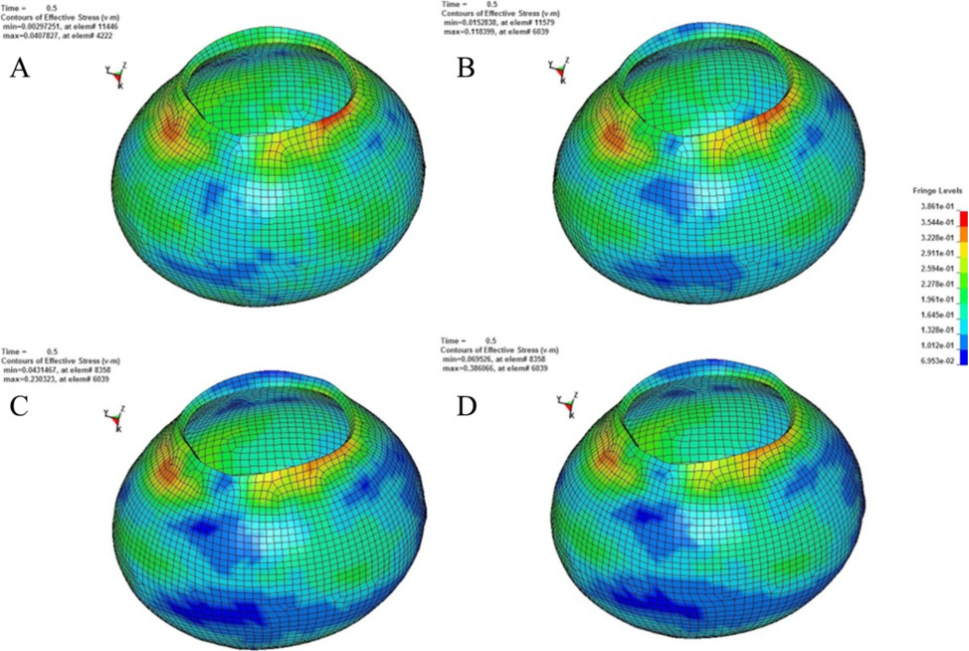
Equivalent stress nephogram of sclera. **a**: 10 mmHg; **b**: 30 mmHg; **c**: 60 mmHg; **d**: 100 mmHg

**Fig. 15:**
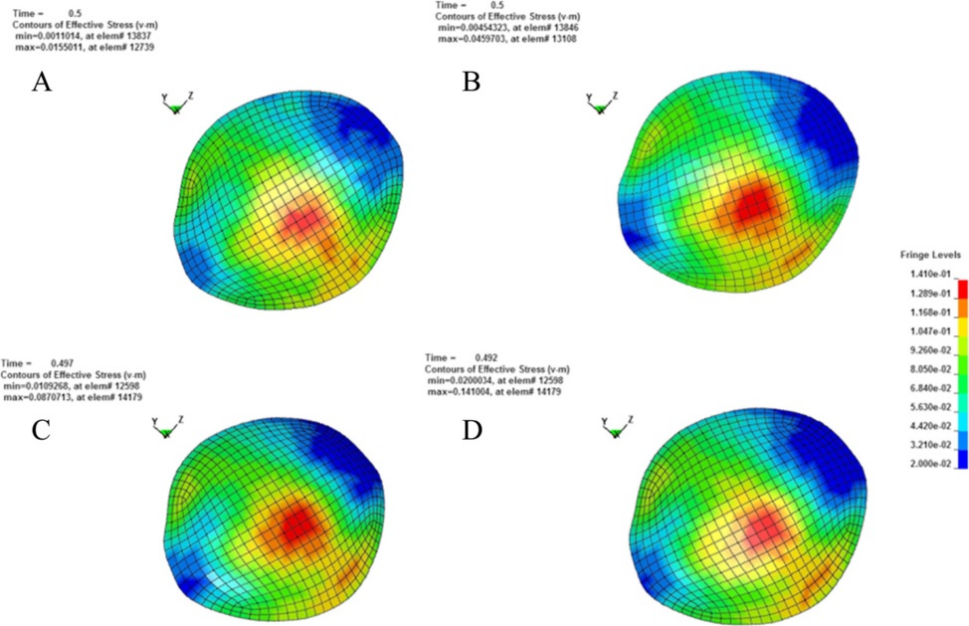
Equivalent stress nephogram of cornea. **a**: 10 mmHg; **b**: 30 mmHg; **c**: 60 mmHg; **d**: 100 mmHg

**Fig. 16:**
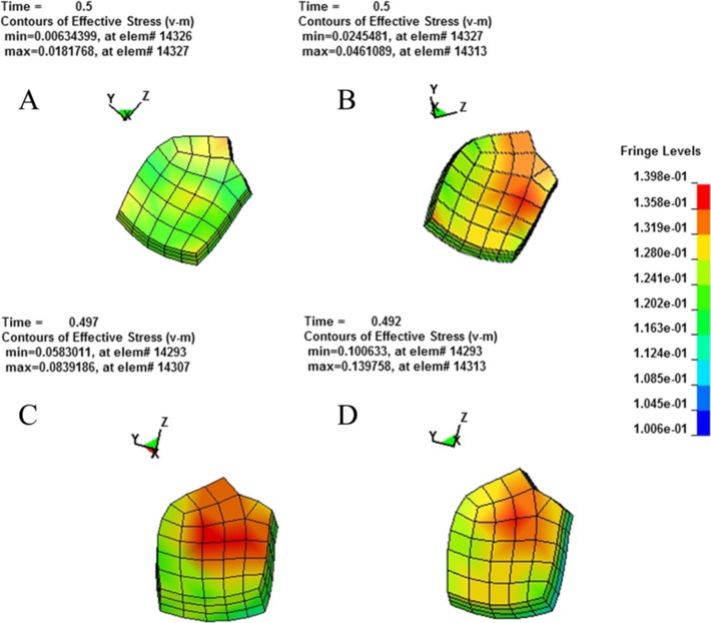
Equivalent stress nephogram of LC. **a**: 10 mmHg; **b**: 30 mmHg; **c**: 60 mmHg; **d**: 100 mmHg

According to the principle of continuum mechanics, we simulated the morphologic strain and mechanical stress of cornea and sclera under different IOP, and obtained the following equivalent strain and stress contours of bovine eye. Figures 9, 10, 11, 12 were the equivalent strain nephogram, while Figs. 13, 14, 15, 16 were the equivalent stress contours. Strain or stress value was increased as the color turned redder, and got closer to zero as color became bluer. By analyzing equivalent stress contours, we found that as simulated IOP was increased, the blue area in the posterior half of sclera expanded while the green area narrowed, indicating that as IOP increased, the stress change in the anterior half of sclera was not obvious while the stress in the posterior half was lowered. By analyzing the corneal equivalent stress nephogram (Fig. 15) we found that no matter how we changed the IOP, the stress of the cornea was always most concentrated in the central part. Moreover, by analyzing equivalent stress contours of LC (Fig. 16), we found that LC stress was mainly concentrated in the central part as IOP was increased.

## Discussion

Acute HIOP glaucoma has some important pathological features such as the damage and atrophy of optic nerve, progressive loss of the RGCs, and so on. It is characterized by the most important clinical symptom-tubular- like visual field. Patients with tubular visual field normally lose peripheral visual field first, which expands to the central field as the disease deteriorates, eventually causing tubular-like visual field. In other words, the visual sensitivity loss and subjective visual sensory defect happens later in the central part of retina than the peripheral part in the progression of glaucoma. Preliminary clinical study showed that RGCs death did not occur at the same time in glaucoma, instead, peripheral RGCs died earlier than the central part [51]. This phenomenon is well known as the selected RGCs death in glaucoma patients [52, 53].

The underlying mechanisms of selected RGCs death in glaucoma have been extensively studied. Some evidences implied that the death of RGC was affected by its size. For example, Quigley *et al.* found that large RGCs were relatively easy to die in glaucoma [10, 54, 55]. However, this argument is controversial as other researchers found that there was no significant difference between the number of lost small and large RGCs examined by retro-tracing RGCs in experimental glaucoma eyes using horseradish peroxidase [53]. Another potential mechanism of selected RGCs death might be related to the expression of functional tyrosine kinase in retinal ganglion cells [12–20], however this hypothesis is contradictory to optic nerve transection model and chronic HIOP model [56, 57]. Other experiments using subunit of glutamate receptor to analyze the selected RGCs death failed to provide promising explanation [58, 59]. Recent studies suggested that neural degeneration in glaucoma was also related to immune system [60–62], and they pointed out that the loss of complement activity in both acquired and innate immune process was involved in RGCs damage [63, 64]. However the roles of the complement system in RGCs damage remain to be further studied. Tong *et al.* studied the selected RGCs death from the perspective of retinal blood supply [21]. Their results showed that the number of lost RGCs in peripheral part was much more than the central part in rats with acute HIOP and its loss rate was obviously related to local blood supply. This result could be explained by the fact that compared with blood vessels in central part, peripheral blood vessel possess a thinner wall and is less elastic, thus prone to developing ischemia by oppression. Although there exist various hypotheses for selected RGCs death, its mechanisms are yet to be further investigated.

Our present experiment established the mathematical FEM of bovine eyeball and studied it from a different perspective. Equivalent stress nephogram of sclera obtained by simulating different IOP (Fig. 14) showed that as simulated IOP increased, the blue area in posterior portion of scleral FEM gradually expanded and in the meantime, the scope of green area gradually narrowed. This result showed that with the gradual increase of IOP, the stress change of anterior half of sclera, which was in front of middle axis of eyeball and approximately represented the areas around retina, was not obvious, while stress on the posterior half of sclera, which approximately represented the middle and central area of retina, becomes smaller. That means, with the increase of simulated IOP, the stress is mainly concentrated in the peripheral part rather than the central or middle part, suggesting that important pressure-bearing tissues such as sclera will deform as IOP increases, leading to corresponding changes of local stress. Such stress can indirectly reflect the pressure from inner side of wall of eyeball. These above results are consistent with Tong's study by establishing mathematical models and enrich his theory by further discussing reasons for selected RGCs death from the perspective of local stress rather than being limited to vascular factors. Another interesting finding from the simulation of increasing IOP by FEM is that, as IOP increased, most stress is concentrated in central part of LC (Fig. 16). This result is similar to Sigal’s [65], showing that optic nerve passing through central part of LC bears the maximum pressure, which is much higher than those taken by peripheral optic nerve. We believe that the scientific significance behind this interesting phenomenon is worth further study.

The results of our FEM simulation of increased IOP bring up another problem worthy of attention. Regardless of how we change the simulative IOP, corneal stress is always most concentrated in the central part (Fig. 15), suggesting that IOP in the central area of cornea is the highest. Clinical researches found that after cutting cornea by laser, as is used in laser-assisted *in situ* Keratomileusis operation for myopia, patients are prone to develop secondary keratoconus [66]. It is generally believed that keratoconus is associated with the thinning of central portion of cornea, which can be verified by our FEM since corneal stress is most concentrated in the central part.

It should be noted that there are some limitations in our study. Firstly, we only studied the i*n vitro* response of the eyeball materials, more *in vivo* biomechanical experiments of eyeball will be studied in our future work. Secondly, the prediction of HIOP in FE modeling simulation need to be validated in *in vivo* experiment, so the clinical value of this FE modeling will be greatly improved.

## Conclusion

As simulative IOP is increased, the stress-concentration scope of the posterior half of sclera gradually narrows, at the same time, the stress-concentration scope on the anterior half of sclera gradually expands, and stress on the LC is mainly concentrated in the central part, suggesting that IOP becomes mainly concentrated in the anterior part of the eyeball as it increases. These findings provide a biomechanical evidence to explain why RGCs in peripheral part die earlier than RGCs in central part under HIOP.
